# Microstructural Analysis and Wear Performance of Carbon-Fiber-Reinforced SiC Composite for Brake Pads

**DOI:** 10.3390/ma10070701

**Published:** 2017-06-26

**Authors:** Goo Byeong-Choon, Cho In-Sik

**Affiliations:** 1 New Transportation Research Department, Korea Railroad Research Institute, Uiwang 16105, Korea; bcgoo@krri.re.kr; 2 Department of Advanced Materials Engineering, Sun Moon University, Asan 31460, Korea

**Keywords:** C/C-SiC composite, microstructure, friction, wear, Raman spectroscopy

## Abstract

Carbon-fiber-reinforced silicon carbide (C/C-SiC) composite is widely used as a friction material owing to its good performance, even though it is more expensive than metallic materials. The light C/C-SiC composite is an ideal candidate for weight reduction of frictional parts. In this study, the friction and wear behavior of C/C-SiC composite was assessed using a ball-on-disk friction tester under dry reciprocating sliding conditions at different temperatures of 25, 100, and 200 °C. The disk specimens were made of C/C-SiC composite, while the mating counterpart pins were made of bearing steel. The microstructure and wear track of the specimens were characterized using a scanning electron microscopy (SEM) and Raman spectroscopy. The microstructural analysis of the wear track revealed that the wear mechanism was abrasive. The friction coefficient and wear behavior of the specimens was dependent on the temperature, where the friction coefficients and wear rate increased with increasing temperature.

## 1. Introduction

Carbon fibers have been widely used as reinforcing elements in high-performance composites used in various industries such as automotive, rail, aerospace, wind energy, marine, sporting goods, etc. [1,2,3]. Carbon fiber reinforced carbon (C/C) composites are often used as primary materials in the fabrication of advanced composites due to high specific elastic modulus, high melting point, enough strength, stiffness and toughness for friction materials. Furthermore, it has been reported earlier that C/C plays a significant role in controlling the friction and wear behavior [4]. However, in harsh applications, where the high resistance to stress and wear at high temperatures is required, it is difficult to improve the tribological characteristics of C/C composites owing to their inert surface property. This may significantly deteriorate the degree of interfacial abrasive in the composite systems [5,6].

To further increase the mechanical properties and to improve the tribological behavior of carbon-fiber-reinforced silicon carbide (C/C-SiC) friction material at high temperatures, one of the most widely accepted methods is adding different kinds of fibers or powders into the matrix as reinforcing elements [7]. In this composite, a Si reacts with the graphite in C/C composite to become a C/C-SiC composite. The presence of ceramic fiber reinforcement plays an important role in controlling the overall surface properties, such as stiffness, strength, thermal stability and tribological properties of the composite as well [8], due to the chemical stability, high hardness and also self-lubricating phenomenon of SiC, which was added to increase the mechanical properties and wear resistance. A C/C-SiC composite usually exhibits higher fracture toughness and lower mechanical properties compared to SiC ceramic, but other excellent properties are retained. The mechanical properties of a C/C-SiC composite significantly depend on the geometry, interphase structure of the fiber and matrix, architecture, etc. The interface between the fiber and matrix controls the mechanical properties, including fracture toughness of the C/C-SiC composite [9].

There are some requirements for C/C composite to be used as a high-performance material. For example, it is required to have a stable friction coefficient, low wear rate for increased life, low cycle cost, and low weight. Moreover, a car needs a rapid dissipation of kinetic energy in the form of heat. Sticking due to heat generation between the pad and disc leads to rapidly accelerated wear and oxidation at the interface. The most important factor among the requirements is friction and wear behavior. A number of investigations on the tribological behavior of the modified composites with fiber reinforcements have been performed to understand their effects on the friction and wear behavior. Earlier studies reported that the added second phase C fiber reinforcement has a significant influence on the wear resistance at room and high temperatures [10]. Nevertheless, drawbacks, such as poor oxidation resistance, high wear rate, low fatigue strength, low hardness, low elastic modulus, and poor interaction with other matrix materials of C/C-SiC composites, limit their tribology-related applications [11].

Rail and vehicle brake friction materials, such as brake pads, discs, and clutches, need to be manufactured from high performance materials with a stable and high friction coefficient, but a low amount of wear [12]. It has been reported that the reduction in porosity and increase in C content in C/C-SiC composite can improve the friction performance and stability at room temperature [13]. However, due to the generation of a high temperature during braking, the friction and wear behavior of C/C-SiC composite at a high-temperature is very important, and studies on the tribological behavior at high temperatures are scarce in the literature. Moreover, most studies on C/C-SiC composite focused on brake discs, rather than brake pads. It is believed that C/C-SiC composite is a good candidate for newly developed pads since hazardous materials, such as Cu, Cr, Zn, and Sb will be restricted from the usage in brake pads in the near future.

The main purpose of this study was to clarify the friction and wear characteristic of C/C-SiC composite in terms of its possibility of being used as a brake pad at high temperatures and to understand wear mechanisms at high temperatures when the composites slide against SAE 52100 bearing steel under dry sliding conditions. The results obtained at high-temperatures were compared with the results that were obtained at room temperature. The results were examined based on the microstructures and examination of the worn surfaces. Raman spectroscopy results were analyzed in terms of C concentration, which was helpful to correlate the microstructure of wear surfaces with tribological performance at high temperatures.

## 2. Experiments

### 2.1. Specimen Preparation

**Figure 1:**
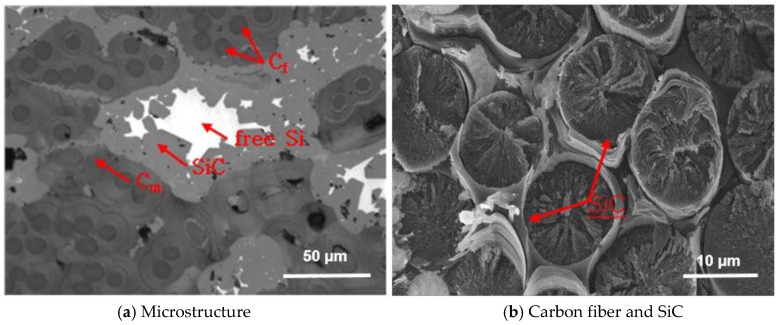
Microstructure of C/C-SiC composite showing the presence of SiC areas in the material. Note: C_f_ is the C fiber, C_m_ is the matrix.

The specimens with dimensions of 10 × 10 × 3 mm^3^ were cut from a C/C-SiC composite. To produce the C/C-SiC composites, carbon fibers coated with phenolic resin were chopped and mixed with phenol resin powder. A preform was produced by hot-forming the mixture at 170 °C for 30 min under pressure. The measured density of the preforms was found to be 1.7 g/cm^3^. The preforms were pyrolyzed in an inert gas ambiance by a heating temperature speed of 10 °C/min up to 1000 °C. The volatile ingredients in the phenol resin began to evaporate at about 200 °C. The final heat treatment of the composite was carried out at 2000 °C. Finally, the C/C composite was infiltrated with molten Si at 1550 °C. The manufacturing process for the C/C-SiC composites was described in more detail in the previous study [14]. The SiC fiber volume fraction in the C/C-SiC composite was about 31%, as shown in Figure 1. The density of C/C-SiC composite was about 2.2 g/cm^3^. The elastic modulus and tensile strength of the specimens were 15 GPa and 60 MPa, respectively. For microstructural characterization, the specimens were polished using an alumina suspension down to a particle size of 1 µm.

### 2.2. Friction and Wear Tests

**Figure 2:**
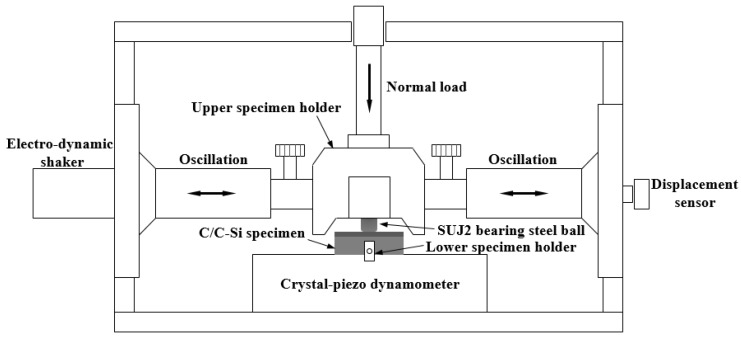
Schematic diagram of the ball-on-disk tribometer.

The friction and wear tests of the specimens were carried out using a ball-on-disk tribometer (Optimol SRV4, Munich, Germany) under dry conditions at different temperatures of 25, 100 and 200 °C. Figure 2 shows a schematic diagram of the tribometer. Table 1 presents the friction and wear test conditions. The calculated Hertzian pressure and circular contact area diameter were found to be in the range of 56–451 MPa and 0.230–0.654 mm, respectively. The lower disk specimen was fixed, while the upper ball oscillated by an electro-dynamic shaker. A bearing steel SAE52100 similar to the brake discs was used as a counter surface with a diameter of 10 mm [15]. All the friction and wear tests were performed three times, and the average values are presented hereafter. Prior to tribological tests, the specimens were cleaned up in deionized water for 10 min to eliminate dust and solid contamination.

### 2.3. Material Characterization

The micro-Vickers surface hardness of the specimens was measured using a hardness tester (Mitutoyo MVK-E3, Mitutoyo Corporation, Sakado, Japan) at a load of 1.962 N for dwell time of 15 s at an approach speed of 60 µm/s. The approach speed was 10 mm/min. At least three measurement results are obtained for each specimen and the average results were reported in this study. A two-dimensional surface profilometer (Mitutoyo SJ-210, Mitutoyo Corporation, Sakado, Japan) was used to measure the average surface roughness (*R_a_*) and wear track profiles; at least three measurement results are obtained for each specimen and the average results were reported in this study. To detect the chemical composition of the specimen, an energy dispersive X-ray spectroscopy (EDX, INCA 350, Oxford Instruments, Oxford, UK) was applied at an accelerating voltage of 20 kV and at a working distance of 3.6 mm. The surface morphology and worn surfaces of the specimens were examined using a scanning electron microscopy (SEM; Nanoeye, SNE-3000M, Suwon, South Korea). For the cross-sectional profiles of wear tracks, at least three measurement results were obtained for each specimen and the average results were reported in this study. Raman spectroscopy is useful to detect a chemical change of C/C-composites before and after friction and wear tests. The microstructure of the specimens was characterized by Raman spectroscopy (LabRam HR, Horiba, Kyoto, Japan) equipped with a green laser of 532 nm in wavelength; size of confocal hole of 200 µm; a power density of 42 MW; a grating of 300 g/mm; numerical aperture (NA) of 0.70 nm; a spot diameter of 1.22 λ/NA; an axial resolution of 0.61 λ/NA. The chemical composition and state of the specimens after the friction and wear test were analyzed by EDS as well. The wear rate of the specimens was defined by the ratio of wear volume to the normal load multiplied by the total reciprocating sliding distance. The wear volume of the disk specimen, *V_disk_*, in dry conditions was calculated using the following relation:

*V_disk_* = *A* × *L* (1)

where *A* is the wear groove area measured using a 2D profilometer, and *L* is the total wear track length. The wear volume of the ball, $V_{ball}$, in the dry conditions was calculated using the following equation:

(2) $$V_{ball}=\frac{\pi h}{6}\sqrt{\frac{3d_{w}^{2}}{4}+h^{2}}$$

where *d_w_* is the mean wear scar diameter, and *h* is the maximum height of the wear scar. h is the parameter related to the mean wear scar diameter by the following equation:

(3) $$h=R-\sqrt{R^{2}-\frac{d_{w}^{2}}{4}}$$

where *R* is the radius of the ball.

The wear rate of the disk specimen and the ball was calculated using the following equation:

(4) $$W=\frac{V}{N\cdot l}$$

where *V* is the wear volume, *N* is the normal load applied, and *l* is the total sliding distance.

## 3. Results and Discussion

### 3.1. Surface Characterizations

**Figure 3:**
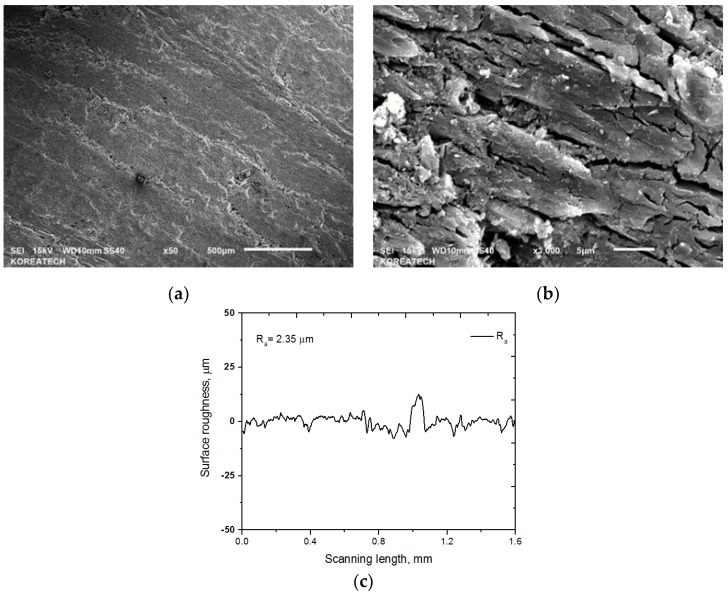
SEM images at low (**a**) and high (**b**) magnification; and cross-sectional surface roughness profile (**c**) of the C/C-SiC composite.

The hardness of material is an important factor in terms of wear resistance. The average surface hardness of the specimens was about 156 HV. The surface microstructure at low and high magnifications along with the cross-sectional surface roughness profile of the specimen is presented in Figure 3. Figure 3a shows that some defects exist on the surface, and the microstructure of the specimen is not homogeneous. Irregular cracks and surface damage in micro-scale are observed, as shown in Figure 3b. These surface defects, cracks or pores deteriorate the mechanical properties, such as strength, hardness, and also the friction and wear behavior of C/C-SiC composites [16]. The most important factor that determines the physical properties of C fiber is the degree of carbonization (C content usually more than 92 in wt %). The average surface roughness of the specimen was 2.35 µm, as shown in Figure 3c. The surface roughness of the specimen is very high, which has the advantage of providing a high friction coefficient during braking. A car needs a rapid dissipation of kinetic energy in the form of heat. Sticking due to heat generation between the pad and disc leads to rapidly accelerated wear and oxidation at the interface.

**Figure 4:**
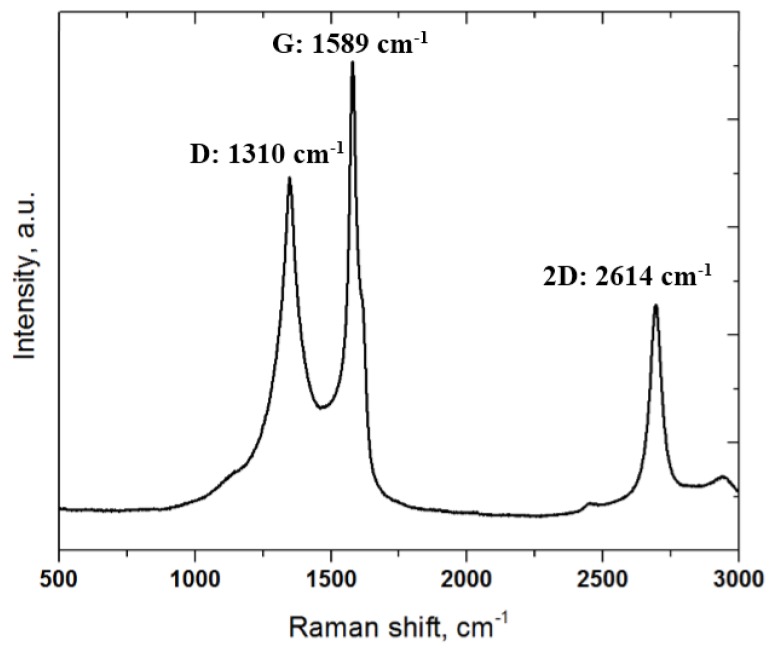
Raman spectrum taken from the C/C-SiC composite.

The Raman spectrum of the specimen is shown in Figure 4. Typical carbon fiber peaks were present in the Raman spectrum of the C/C composite, as shown in Figure 4 [17]. The peak at around 1590 cm^−1^ (G band) with a shoulder around 1604 cm^−1^ was assigned to the in-plane vibration of the C-C bond (G band), which is typical of defective graphite-like materials, and the peak at around 1310 cm^−1^ (D band) was activated by the presence of disorder in the carbon systems. The Raman spectrum showed another peak at around 2610 cm^−1^ (2D band), which was attributed to the overtone of the D band [17].

### 3.2. Friction and Wear Behavior

**Figure 5:**
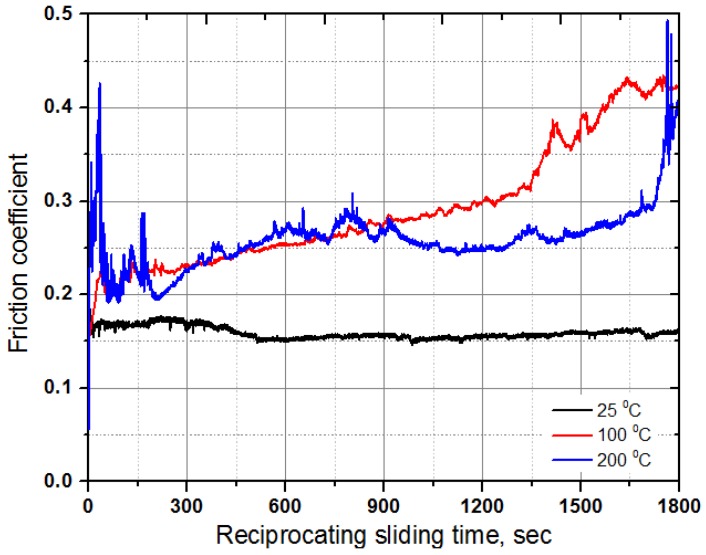
Variation in friction coefficient of the C/C-SiC composite with respect to time at temperatures of 25, 100 and 200 °C.

**Figure 6:**
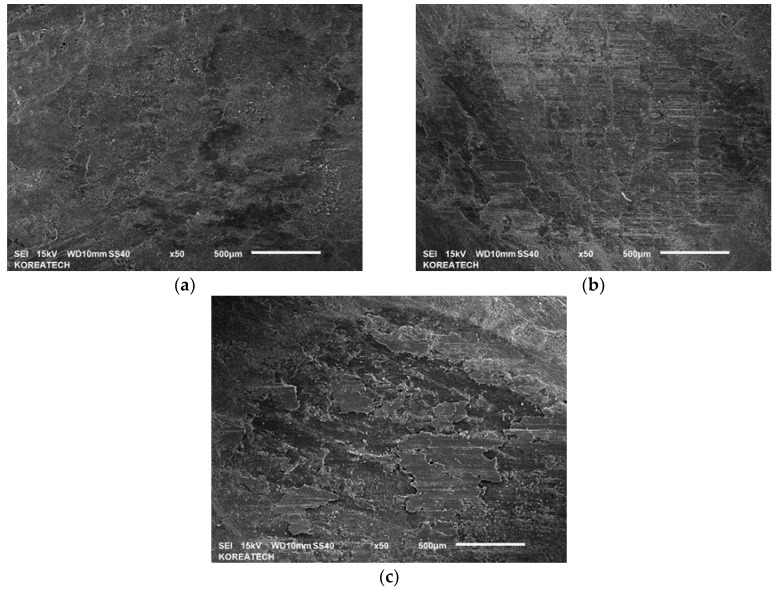
SEM images of wear track formed on the C/C-SiC composite at temperatures of 25 °C (**a**); 100 °C (**b**) and 200 °C (**c**).

**Figure 7:**
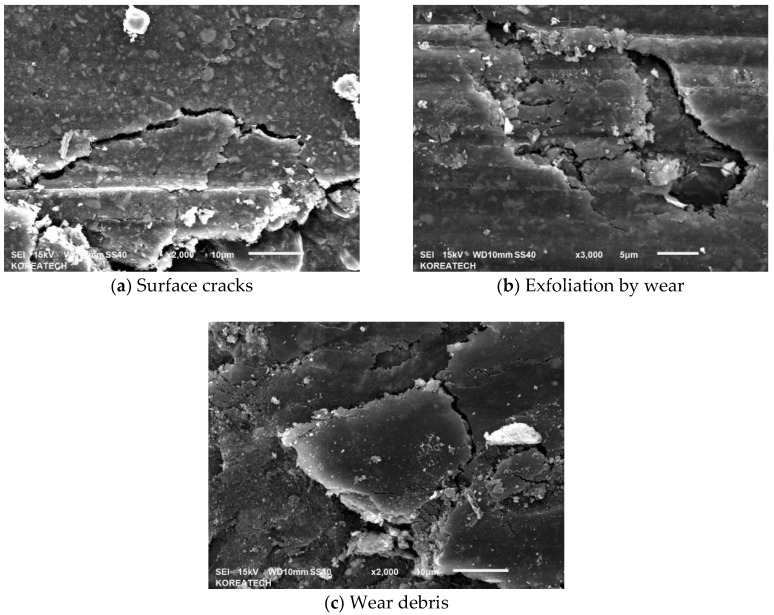
SEM images of wear tracks of the C/C-SiC composite showing the cracks on the surface.

**Figure 8:**
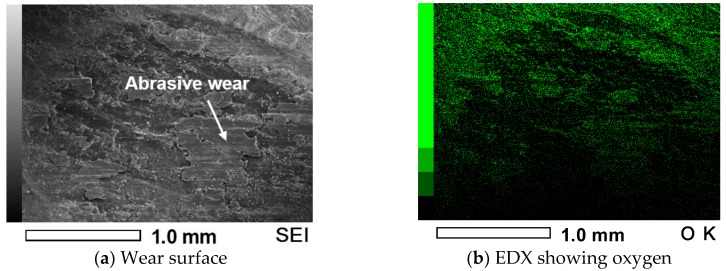
SEM image of wear track formed on the C/C-SiC composite at temperature 200 °C showing the wear mechanism along with oxidation state.

Friction brakes decelerate a car by dissipating the kinetic energy of a running car into frictional heat, which has a significant influence on the behavior of the brake disc and pads. Figure 5 shows the variation in friction coefficients for the specimen at temperatures 25, 100 and 200 °C. At a temperature of 25 °C, a very stable frictional behavior with a value of 0.16–0.17 was observed. However, the friction coefficient increased gradually with the sliding distance, and reached about 0.42 after a sliding time of 30 min at a temperature of 100 °C. Interestingly, the friction coefficient was very high at the initial stage of the test, and then the friction coefficient decreased drastically and increased gradually at a temperature of 200 °C. After a sliding time of about 15 min, the friction coefficient stabilized and decreased a bit, and then it started to increase and reached a very high friction coefficient value of about 0.48–0.50 at the end of the test. Sudden reduction and increase with sliding time in friction coefficient value, which was lower than the friction coefficient at a temperature of 100 °C, may be attributed to the smoothing phenomenon at the contact interface and to the presence of wear-induced particles and debris at the contact interface during reciprocating sliding under dry conditions. This study clearly demonstrated that the friction coefficient of the specimens increased with temperatures from about 0.18 to 0.48. The main reason for the increase in friction coefficient with an increasing temperature may be the decomposition of the composite. Similar results were reported in a previous study where the friction coefficient increased as the friction temperature rose from 100 to 300 °C [18]. The friction and wear behavior can be explained in terms of plasticity of the composite at different temperatures [19] because C/C-SiC composites soften with increasing temperature under dry conditions where hard phase SiC cannot be cut by shearing and easily break. SEM was employed to observe the surface of worn out specimens as shown in Figure 6. It can be seen that after sliding under dry conditions at temperatures of 25, 100 and 200 °C, the specimens were severely worn out. The specimen tested at a temperature of 25 °C showed relatively minor wear compared to the specimens that were tested at temperatures 100 and 200 °C. This kind of wear behavior is in a good agreement with the friction coefficient presented in Figure 5. It signifies that the resistance to wear of the composite was weakened with a temperature increase. Surface damage after friction and wear tests at different temperatures are presented in Figure 7. All the surface specimens were broken up with cracks. The wear mechanism for all the specimens was found to be abrasive mode, where the surface layers were peeled off gradually layer-by-layer as shown in Figure 8. Abrasive wear is usually considered as a primary wear mechanism for composite materials [4]. An oxidative wear mode was also observed and was determined from the SEM images and EDX patterns of the worn out surfaces.

**Figure 9:**
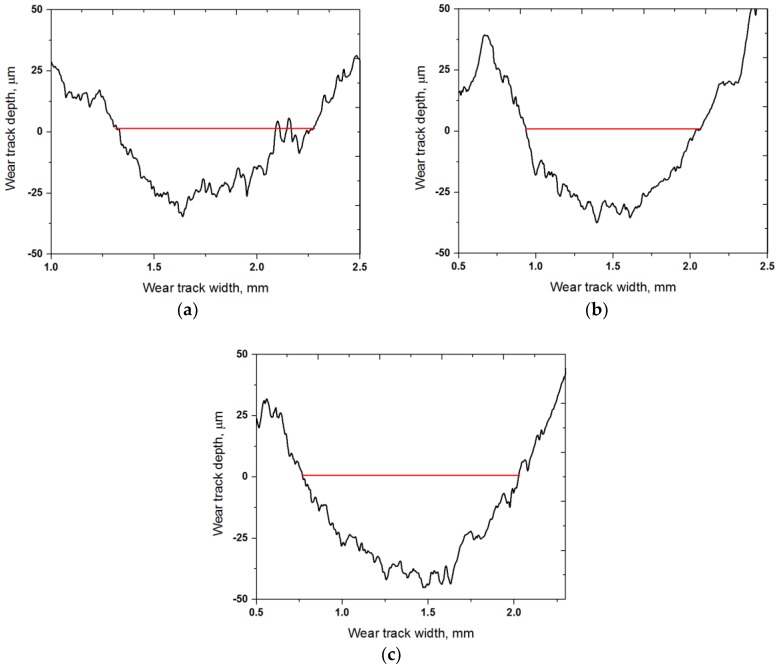
Cross-sectional profiles of wear track formed on the C/C-SiC composite slid against AISI52100 steel at temperatures of 25 °C (**a**); 100 °C (**b**) and 200 °C (**c**).

**Figure 10:**
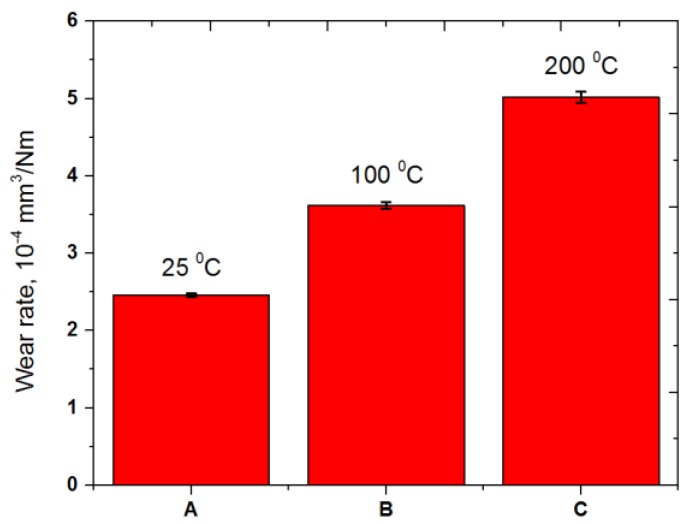
Wear rate of the C/C-SiC composite slid against AISI52100 steel at temperatures of 25, 100, and 200 °C.

Figure 9 presents the cross-sectional wear track profiles of the specimens tested at temperatures 25, 100, and 200 °C. The friction and wear results revealed that the specimen tested at a temperature of 25 °C exhibited a shallower and narrower wear track. The depth of the wear track of the specimens tested at temperatures 25, 100, and 200 °C was about 31, 38 and 46 μm, respectively. The depth and diameter of the wear track increased with an increasing temperature, as shown in Figure 9b, c. These cross-sectional profiles allowed us to calculate the wear rate of the specimens. Figure 10 shows the wear rates at temperatures 25, 100, and 200 °C. The wear rate increased with an increasing temperature.

**Figure 11:**
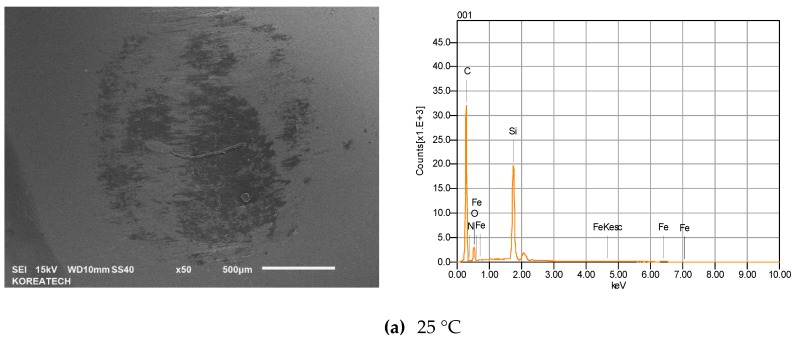

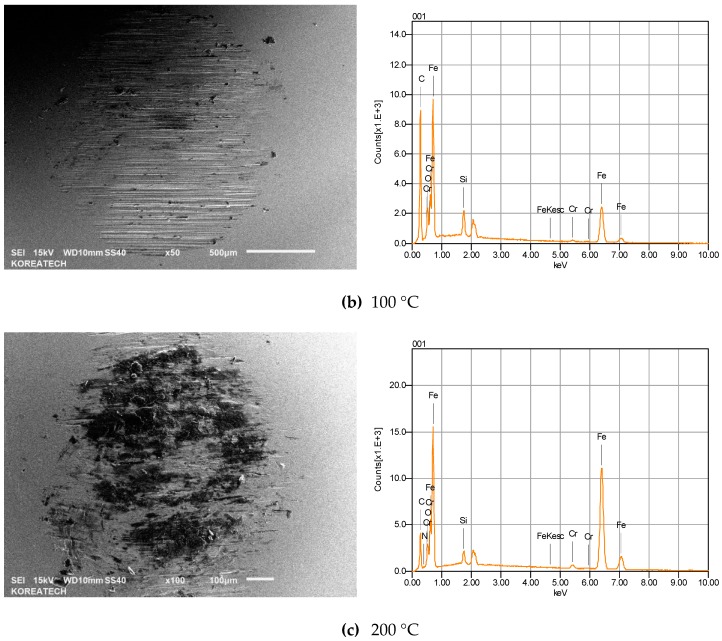
SEM images and EDS patterns of wear scars formed on the AISI52100 steel ball slid against the C/C-SiC composite at temperatures of 25, 100 and 200 °C.

**Figure 12:**
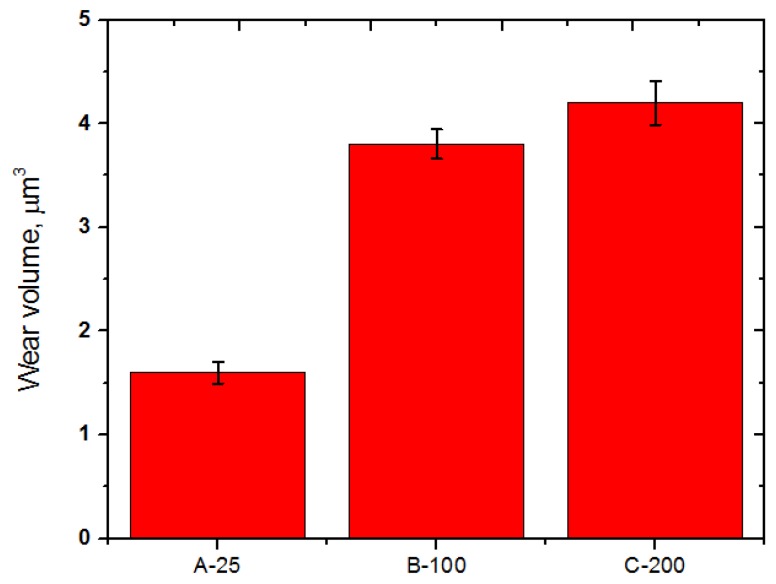
Wear volume of AISI52100 steel ball slid against the C/C-SiC composite at temperatures of 25, 100 and 200 °C.

Figure 11 presents the wear scar formed on the counter ball surfaces and its chemical composition when the balls slid against the disc specimens at temperatures of 25, 100, and 200 °C. No significant difference in wear scar was found for the balls tested at temperatures of 25 and 100 °C. However, the counter ball surface tested at 200 °C demonstrated a much larger wear scar diameter and rougher surface with a high amount of Fe and Cr elements, which were transferred from the counter surface, than the specimen tested at a temperature of 25 °C. This means that the contact area of the specimen tested at a temperature of 25 °C with the corresponding ball was smaller than that of the specimens tested at a temperatures of 200 °C with the corresponding ball. Figure 12 shows the wear volume of the balls. The wear volume was quantified using the equation presented above. It is obvious that the ball tested at a temperature of 25 °C exhibited a higher resistance to wear than the specimens tested at a temperature of 200 °C. A similar result was reported in [20]. The presence of SiC in a C/C composite increases the friction coefficient during braking, but the wear resistance can also be mitigated because of the abrasive character of SiC. With an increasing temperature, the friction coefficient and wear rate deteriorated due to the flexion by debonding of SiC grains where the C fiber loosened. Braking increases the temperature of the disc and the pads, and the friction coefficient. The wear rate also increases simultaneously. In addition, the friction coefficient of a C/C-SiC composite during braking depends on the surface hardness, which increases the friction coefficient due to the ploughing effect [21]. Softening occurs in the friction surface due to heat generation during braking, and relatively hard SiC particles in the C/C composite come into hard contact with the counter surface. Either the material of the disc or the pad has an influence on the friction coefficient of a frictional brake. A number of factors affect the friction coefficient and wear rate of a C/C-SiC composite. For example, the pyrolytic C amount, porosity and SiC content as well as braking pressure and speed play an important role in controlling the friction and wear behavior of a C/C-SiC composite.

**Figure 13:**
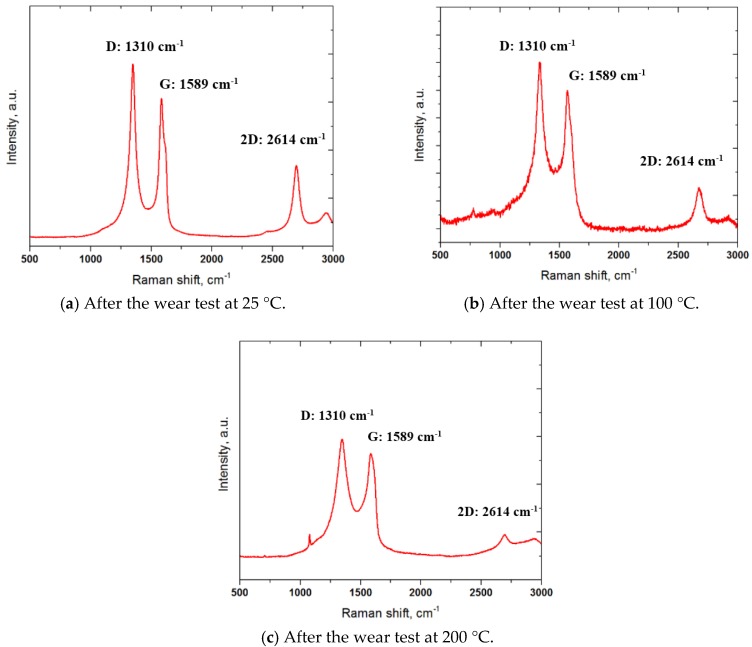
Raman spectra obtained from wear track formed on the surface of the C/C-SiC composite slid against AISI52100 steel at temperatures 25, 100 and 200 °C.

The friction and wear behavior of a C/C-SiC composite depends very much on the microstructure and surface properties. Graphitization of a C/C-SiC composite has an important influence on the friction and wear behavior of a C/C-SiC composite at the initial stage of braking. In this regard, the microstructure of the C/C-SiC composite after friction and wear test was characterized by Raman spectroscopy. Figure 13 shows the Raman spectroscopy results taken from the worn surfaces at temperatures of 25, 100, and 200 °C. The intensity of C-C bonds (G band) that was tetrahedral (sp^3^) was found to be lower than the *D band* after friction tests at temperatures of 25, 100, and 200 °C. These changes may be due to the C concentration after wear tests, and account for the oxidation as detected by EDS in Figure 11. It can be seen that the intensity of the *2D band* decreased with an increasing temperature, which was also due to the role of C concentration [22]. At temperatures of 100 and 200 °C, the friction and wear increased and the C concentration started to increase due to the high wear of the bearing steel, which resulted in a reduction in Raman intensity due to the increase in C concentration. Moreover, some new peaks appeared at about 750 and 1100 cm^−1^ at temperatures of 100 and 200 °C (see Figure 12b, c), respectively. These peaks may be attributed to the presence of SiC and Si elements formed at the contact interface and graphitization during sliding under dry conditions. It has been reported that the friction coefficient of C/C-SiC composites with no graphitization is higher than the friction coefficient of C/C-SiC composites with graphitization. It is also observed that graphite can act as a solid lubricant to reduce the friction coefficient at the initial stage of braking and normalize the fluctuation in the friction coefficient in the final stage of braking. Moreover, graphitization may increase the content of SiC in C/C-SiC composites, where graphite crystallite can be formed [23], which can lead to softening of C/C-SiC composites. A precise description of the evaluation of graphite spectrum with consideration of amorphization or graphitization of the structure was reported earlier [24,25]. In particular, the G band intensity is used as a normalized intensity to study the evolution of the G band by the calculation of the ratio ID/IG.

## 4. Conclusions

The friction and wear behavior of C/C-SiC against AISI 52100 bearing steel ball was investigated using a ball-on-disk tribometer under dry conditions at different temperatures. The following conclusions may be drawn from the experimental results:

- The average surface roughness of the C/C-SiC specimen before friction and wear tests was found to be 2.35 µm, while the surface hardness was found to be 156 HV. However, it should be noted that the surface roughness values highly depends on the location of the composite due to the presence of pores on the surface.
- The friction coefficient of the C/C-SiC specimen at 25 °C was very stable with a value of about 0.16, while the friction coefficients at 100 and 200 °C were unstable. The friction coefficient increased with an increasing temperature in the early period of the test, but after some sliding the friction coefficient at 200 °C was lower than that at 100 °C.
- The wear resistance decreased with an increasing temperature. The wear mechanism of C/C-SiC against SAE52100 steel was revealed to be abrasive mode.
- Raman spectroscopy results revealed that the intensity of the G band was not changed significantly for all the specimens after the friction and wear tests. However, the intensity of D and 2D bands decreased with an increasing temperature.
- It is believed that the developed C/C-SiC composite can be a good candidate for an eco-friendly brake pad.

## Figures and Tables

**Table 1:** Friction and wear test conditions.

Normal Load (N)	Frequency (Hz)	Stroke (mm)	Sliding Time (min)	Temperature (°C)	RH (%)
50	10	1	30	25, 100, 200	60
